# Graphical analyses in delay interaction networks

**DOI:** 10.1186/1471-2202-14-S1-P413

**Published:** 2013-07-08

**Authors:** Patricia Wollstadt, Raul Vicente, Michael Wibral

**Affiliations:** 1 MEG Unit - Brain Imaging Center, Goethe University, Frankfurt,60528, Germany; 2 Frankfurt Institute for Advanced Studies (FIAS), Goethe University, Frankfurt,60528, Germany

## 

Network or graph theory has become a popular tool to represent and analyze large-scale interaction patterns in the brain. To derive a functional network representation from experimentally recorded neural time series one has to identify the structure of the interactions between these time series. In neuroscience, this is often done by pairwise bivariate analysis because a fully multivariate treatment is typically not possible due to limited data and excessive computational cost. Furthermore, a true multivariate analysis would consist of the analysis of the combined effects, including information theoretic synergies and redundancies, of all possible subsets of network components. Since the number of these subsets is the power set of the network components, this leads to a combinatorial explosion (i.e. a problem that is computationally intractable). In contrast, a pairwise bivariate analysis of interactions is typically feasible but introduces the possibility of false detection of spurious interactions between network components, especially due to cascade and common drive effects. These spurious connections in a network representation may introduce a bias to subsequently computed graph theoretical measures (e.g. clustering coefficient or centrality) as these measures depend on the reliability of the graph representation from which they are computed. Strictly speaking, graph theoretical measures are meaningful only if the underlying graph structure can be guaranteed to consist of one type of connections only, i.e. connections in the graph are guaranteed to be non-spurious.

We propose an approximate solution to improve this situation in the form of an algorithm that flags potentially spurious edges that are due to cascade effects and "three node" common drive effects in a network representation of bivariately analyzed interactions. As these two effects are responsible for a large part of spurious connections in bivariate analyses, their removal would mean a significant improvement of the network representation over existing bivariate solutions.

Our approach is based on the detection of directed interactions and the weighting of these interactions by their reconstructed interaction delays. We demonstrate how both questions can be addressed using a modified estimator of transfer entropy (TE). TE is an implementation of Wiener's principle of observational causality based on information theory [1], and detects arbitrary linear and non-linear interactions. Using a modified TE estimator that uses delayed states of the driving system, one can mathematically prove that transfer entropy values peak if the delay of the state of the driving system equals the true interaction delay [2]. From this analysis, we derive a delay weighted network representation of directed interactions. On this network representation, potentially spurious interactions can be detected by analyzing sets of alternative paths between two endpoints in terms of their summed delays. The proposed algorithm may be used to prune spurious edges from the network, improving the reliability of the network representation itself and enhancing the applicability of subsequent graph theoretical measures. For the detection of "multi-node" common drive effects, that are not considered in this study, a theoretical solution exists as well, extending the power of the method, but this solution has not been implemented yet.

We demonstrate the application of this algorithm to networks of interacting neural sources in magneto-encephalographic data, and show that roughly 30% of bivariate interactions in these data are potentially spurious, and thus alter graph properties.

We conclude that the post hoc correction provided by our approach is a computationally less demanding alternative to a fully multivariate analysis of directed interactions, and preferable in cases were a multivariate treatment of the data is difficult due to the limited amount of data available.

